# Omega-3 polyunsaturated fatty acids are associated with microbiota-related 18β-glycyrrhetinic acid alterations and M2 macrophage polarization in type 1 diabetes mellitus

**DOI:** 10.3389/fphar.2026.1871892

**Published:** 2026-06-17

**Authors:** Yifan Guo, Fang Hu, Allan Zijian Zhao, Yingjuan Zeng, Haoying Chen, Ai Wang, Xiaoxi Li, Li Cong

**Affiliations:** 1 Department of Endocrinology and Metabolism, The Fifth Affiliated Hospital, Sun Yat-sen University, Zhuhai, Guangdong, China; 2 Department of Endocrinology and Metabolism, Shunde Hospital of Southern Medical University, Guangzhou, China; 3 Department of Immunology, Nanjing Medical University, Nanjing, China

**Keywords:** gut micribiota, macrophag, mult omics, omega-3 poly unsaturated fatty acids, type 1 daibetes mellitus

## Abstract

**Introduction:**

Omega-3 polyunsaturated fatty acids (PUFAs) have been widely reported to exert beneficial effects in type 1 diabetes mellitus (T1DM). However, the mechanisms by which Omega-3 PUFAs influence pancreatic islet function through the gut-islet axis remain incompletely understood. This study aimed to investigate whether Omega-3 PUFAs-associated alterations in gut microbiota composition and their metabolites are involved in the modulation of the pancreatic islet microenvironment in T1DM, and to explore the potential immunological mechanisms underlying these effects.

**Methods:**

Fecal microbiota transplantation (FMT) was performed to evaluate the effects of Omega-3 PUFAs-associated gut microbiota in non-obese diabetic (NOD) mice. Immune cell composition and inflammatory status within pancreatic islets were analyzed using transcriptomic profiling, flow cytometry, and immunohistochemistry. Metabolomics analysis was performed to investigate the association among Omega-3 PUFAs, gut microbiota, and microbiota-related metabolites. *In vitro* co-culture systems were further established to evaluate the effects of selected metabolites on macrophage polarization and insulin production and secretion in pancreatic β-cells.

**Results:**

Omega-3 PUFAs treatment and FMT were associated with reduced islet inflammation and a marked enrichment of the *Eubacterium coprostanoligenes group* (*E. coprostanoligenes*). Enhanced M2 macrophage polarization was observed in the islet microenvironment of FMT mice. Among gut microbiota metabolites, 18β-glycyrrhetinic acid (18β-GA) showed a strong association with *E. coprostanoligenes*, and *in vitro* experiments suggested a potential involvement of *E. coprostanoligenes* in the metabolic conversion process related to 18β-GA. In co-culture systems, 18β-GA promoted macrophage polarization toward an M2-like phenotype, which was accompanied by increased insulin production and secretion in pancreatic β-cells.

**Conclusion:**

These findings suggest that Omega-3 PUFAs-associated alterations in gut microbiota composition and 18β-GA-related metabolic changes may contribute to the modulation of the pancreatic immune microenvironment and β-cells function in T1DM. This study provides additional insights into lipid-microbiota-immune interactions relevant to T1DM and supports further investigation of microbiota-associated immune regulation.

## 1 Introduction

Type 1 diabetes mellitus (T1DM) is a chronic autoimmune disease characterized by the immune-mediated destruction of pancreatic β-cells, resulting in lifelong dependence on insulin injections and an increased risk of severe complications, including diabetic ketoacidosis (Bottazzo et al., 1985; Gepts, 1976; Syed, 2022). Genes (such as the HLA haplotypes) conferring susceptibility are highly important in T1DM, but environmental factors are increasingly recognized as having an important role in driving the onset and development of disease (Groele and Szypowska, 2023). The gut microbiota plays an important role in systemic immunity by modulating pro-inflammatory immune responses and has a central role in maintaining the balance of immunoregulatory responses (Gasmi et al., 2021). Dysregulation of the gut microbial ecosystem (dysbiosis) leads to failure in intestinal barrier function, enhanced peripheral endotoxemia, and systemic low-grade chronic inflammation (Wang G. et al., 2024). This results in the unmasking of autoreactive T Cells and the targeting of pancreatic islets. These gut-islet interactions provide an exciting possibility for therapeutic intervention in T1DM.

Omega-3 polyunsaturated fatty acids (PUFAs) (e.g., eicosapentaenoic acid [EPA] and docosahexaenoic acid [DHA]) are well-known dietary anti-inflammatory fatty acids. Clinical and preclinical evidence showed that Omega-3 PUFAs attenuate systemic inflammation, lower serum triglycerides and induce resolution of inflammation by making specialised pro-resolving mediators (Valenzuela et al., 2024; Videla et al., 2022). Some studies, including our previous work, have reported beneficial associations between Omega-3 PUFAs intake and T1DM-related outcomes (Bi et al., 2017).

In addition, Omega-3 PUFAs supplementation markedly changed the abundance of gut microbiota, increasing the abundance of beneficial microbial taxa such as *Lactobacillus* and *Ruminococcus*, improved intestinal barrier integrity and reduced serum concentrations of pro-inflammatory cytokines such as IL-1β and TNF-α (Zhu et al., 2021; Fu et al., 2021). However, how Omega-3 PUFAs modulate islet-specific autoimmunity is not completely clear. Crucially, it is unknown whether the protective effect of Omega-3 PUFAs on pancreatic β-cells acts directly on the host in metabolic ways, or rather indirectly through altered gut microbiota and its metabolites.

Recent research has highlighted the role of gut microbiota in modulating autoimmunity in both the intestine and pancreatic islets in T1DM (Atkinson and Chervonsky, 2012; Adhikary et al., 2024a). Alterations in gut microbiota composition have been observed in pediatric patients at the onset of T1DM compared with healthy controls (Biassoni et al., 2020), and modulation of gut microbiota has been shown to improve glycemic parameters in experimental models of T1DM (Chen et al., 2022). Our previous research, along with several other studies, has shown that Omega-3 PUFAs can directly influence the diversity and abundance of gut microbiota, thereby restoring gut barrier integrity (Parolini, 2019). Furthermore, studies have indicated that non-obese diabetic (NOD) mice fed diets enriched with soluble fiber inulin and Omega-3PUFAs exhibited an increase in beneficial bacterial species (Lo Conte et al., 2022). Notably, emerging clinical evidence suggests that fecal microbiota transplantation (FMT) may slow disease progression in individuals with new-onset T1DM (de Groot et al., 2021). Given its immunomodulatory capacity (Wastyk et al., 2021), the gut microbiota is a promising therapeutic target for treating T1DM (Calcinaro et al., 2005; de Groot et al., 2021). Nevertheless, the lack of studies performing FMT combined with multiomics analysis, for example, metabolomics and transcriptomics, impedes the identification of precise mechanisms of microbial-host cross-talk that could be therapeutic targets (Wang C. et al., 2024). Hence, the potential relevance of microbiota-based approaches, including prebiotics, probiotics or specific microbiota-derived metabolites, in modulating immune and inflammatory features related to T1DM remains incompletely understood.

At present, these gaps are partially addressed by the present study to test whether an Omega-3 PUFAs-induced protection of pancreatic β-cells in NOD mice is mediated causally by the gut microbiota. For this purpose, we designed a comprehensive experimental study combining dietary Omega-3 PUFAs supplementation with FMT from Omega-3 PUFAs-treated donors. Multiple approaches were employed, including 16S rRNA sequencing to characterize gut microbial composition, metabolomics, pancreatic islet transcriptomics, flow cytometry, immunohistochemistry, and *in vitro* co-culture systems of macrophages and pancreatic β-cell lines. This powerful combination of tools enabled us to explore the hypothesis that specific gut microbiota, induced by Omega-3 PUFAs, harbor bioactive metabolites capable of shifting the islet immune microenvironment from a pro-inflammatory state toward an immunoregulatory phenotype, thereby promoting β-cell survival.

Objectives of this study were the following three main points: first, to identify whether Omega-3 PUFAs change the gut microbiota composition and decrease the islet inflammation in NOD mice; second, to investigate whether FMT from Omega-3 PUFAs treated donors could transfer microbiota-associated immune and inflammatory features to recipient mice; third, to identify the specific microbiota or microbial metabolite potentially associated with the observed immunomodulation effects. Specifically, we aimed to validate the effects of the microbial metabolite 18β-glycyrrhetinic acid (18β-GA) in promoting an anti-inflammatory M2-like macrophage phenotype and the maintenance of β-cell insulin-producing ability. By clarifying the gut-islet axis with causal mechanisms, our study provides a basis for the development of microbiota- or metabolite-based interventions for the prevention and treatment of T1DM.

## 2 Materials and methods

### 2.1 Study design

This study was designed as a controlled exploratory animal experiment to investigate the effects of Omega-3 PUFAs and gut microbiota transplantation on islet inflammation and β-cell function in NOD mice. 22 female NOD/LtJGpt mice (3-4 weeks old) were randomly allocated into experimental groups using simple random selection during ear-tagging. Female NOD mice were selected due to their higher and more consistent incidence of spontaneous T1DM compared with male NOD mice, which provides a stable model for mechanistic investigation.

Specifically, 10 mice were assigned to two dietary intervention groups (n = 5): a semi-purified control diet group (Supplementary Table S1) and an Omega-3 PUFAs diet group supplemented with 10% fish oil (ShangHai HOPE Industry Co., Ltd.), following our previous experimental protocol (Bi et al., 2017). An additional 12 mice were assigned to two fecal microbiota transplantation (FMT) groups (n = 6) and fed with semi-purified control diet. Fresh fecal transplants were pooled separately from donor mice fed the control diet and Omega-3 PUFAs diet, and administered to the recipient mice for 1 week, respectively.

The primary outcome measure was pancreatic islet inflammation, assessed by histological insulitis scoring. The sample size was based on prior publications and practical feasibility. No power calculation was performed, and five to six animals per group were used to balance scientific validity and ethical animal use.

A total of 22 NOD mice completed the study and were included in the analysis. No *a priori* exclusion criteria were set, and no animals or data points were excluded.

Investigators were aware of the group allocation during the allocation and conduct of the experiment. However, outcome assessments and data analyses, including histological scoring and flow cytometry analysis, were performed by investigators blinded to group assignments to minimize potential assessment bias.

### 2.2 Mice and diets

The animal studies were pre-approved in advance by the Ethics Committee of The Fifth Affiliated Hospital, Sun Yat-sen University and all ethical obligations were com-plied with (animal ethics approval No.00211). Female NOD/LtJGpt mice, 3-4 weeks, were obtained from GemPharmatech Co., Ltd. (Jiangsu, China) and housed under strict specific pathogen-free (SPF) conditions. All mice were maintained in a controlled environment with a 12-h light/dark cycle. Mice were acclimated for 1 week after arrival before the start of dietary or FMT interventions. Mice were anesthetized with isoflurane inhalation (induction: 3%-4%; maintenance: 1.5%–2% in oxygen) before samples collection. At the experimental endpoint, mice were euthanized by cervical dislocation under deep anesthesia to minimize suffering. Each individual mouse was a single experimental unit.

Dietary interventions consisted of a semi-purified control diet or the same control diet supplemented with 10% fish oil (Omega-3 PUFAs diet). Two types of SPF-grade mouse diets were produced by XIETONG Bioengineering Co., Ltd.

Mice from each group were housed in separate cages, which were rotated weekly within the animal facility to avoid location bias, and measurements were performed according to practical convenience.

### 2.3 Bacterial culture

*Eubacterium coprostanoligenes* (*Eubacterium coprostanoligenes*, ATCC 51222) was purchased from the Mingzhoubio (Ningbo, China) and cultured in Chopped Meat Carbohydrate (CMC) broth (Mingzhou, Ningbo, China) supplemented with chopped beef particles (10 g per 1 L broth), hemin chloride (5 mg per 1 L broth) and vitamin K1 (50 mg per 1 L broth). Cultures were incubated anaerobically at 37 °C in an anaerobic chamber (85% N_2_, 10% CO_2_, 5% H_2_).

To verify that 18β-GA is derived from *E. coprostanoligenes*, three groups were established in parallel (n = 5): (1) CMC broth (without glycyrrhizic acid (GL, MCE, USA) or *E. coprostanoligenes*), (2) CMC broth + GL (without bacteria), (3) CMC broth + *E. coprostanoligenes* + GL. GL was added to the cultures at a final concentration of 200 μM, and incubated for up to 24 h.

### 2.4 Cell culture

RAW 264.7 cells were provided by Servicebio (Wuhan, China) and cultured in DMEM (Gibco, USA). The NIT-1 pancreatic β-cells were provided by Whelab (Shanghai, China) and grown in Ham’s F12 K medium (Icellbioscience, Shanghai, China). Both media were enriched with 10% fetal bovine serum (VivaCell, Shanghai, China) and 1% penicillin-streptomycin solution (Gibco, USA). The cells were then cultured under standard conditions (at 37 °C and supplemented with 5% CO_2_) until they reached approximately 80% confluence.

To induce polarization, RAW264.7 cells were exposed to lipopolysaccharide (LPS, Sigma, USA) at a concentration of 1 μg/mL for 6 h to promote M1 polarization. Simultaneously, 18β-GA (Aladdin, Shanghai, China) was administered for 24 h to evaluate its effects on macrophage polarization.

To investigate the interactions between macrophages and pancreatic β-cells, a 12-well transwell co-culture system (0.4 μm pore size; Corning) was used. This system allowed soluble factors to diffuse between the two cell types while preventing direct cell-to-cell contact. First, after pretreatment with or without LPS and 18β-GA, RAW264.7 cells were seeded in the upper chamber of the transwell insert, while NIT-1 cells were seeded in the lower chamber. Due to the higher glucose content of DMEM, which stimulates insulin secretion in NIT-1 cells, both the upper and lower chambers were switched to fresh DMEM on the day of co-culture. After 48 h of incubation, the supernatant was collected to assess insulin levels, and NIT-1 cells were processed for immunofluorescence (IF).

### 2.5 Glucose tolerance test

The intraperitoneal glucose tolerance test (IPGTT) was performed as previously described (Bi et al., 2017). Briefly, mice were fasted for 16 h prior to the experiment. Subsequently, each mouse received an intraperitoneal injection of glucose (2 g/kg of body weight; Otsuka, Japan). Blood glucose levels were recorded at baseline (0 min) and at 15, 30, 60, 90 and 120 mins post-injection using an automatic glucose monitor (Roche, Switzerland) with blood obtained from a tail snip.

### 2.6 Histomorphological analysis of insulitis

After the mice were euthanized, their pancreases were collected and fixed overnight in 4% paraformaldehyde. The fixed tissues were then embedded in paraffin wax, and 4 μm-thick sections were prepared. These sections were stained with hematoxylin and eosin to assess islet insulitis. Slides were scanned using the Pannoramic Flash P250 Scanner (3DHistech, Hungary). Insulitis was scored on a 4 levels and shown as follows: 0) no insulitis; 1) peri-insulitis (leukocytes surrounding the islet); 2) invasive insulitis (25%–50% islet infiltration); and 3) severe insulitis (more than 50% infiltration). To ensure unbiased evaluation, the scoring pathologist was blinded to the group assignments.

### 2.7 16s rRNA sequencing and analysis

Fecal pellets were collected in sterile 1.5 mL tubes and stored at −80 °C. Total microbial genomic DNA was extracted using the MagPure Soil DNA LQ Kit (Magan, China). Samples from the control and Omega-3 groups were sequenced by Guangzhou GENE *Denovo* (Guangzhou, China) on the Illumina PE250 platform. The V3–V4 regions of the 16S rRNA gene were amplified using primers 341F and 806R. Fecal samples from the Con-FMT and Omega3-FMT groups were sequenced by Oebiotec (Shanghai, China) using the Illumina NovaSeq 6,000 platform, with primers 515F and 907R targeting the V4–V5 regions. Sequence analyses were performed using QIIME2. Alpha diversity was evaluated using the Chao1 and Shannon indices (Chao and Bunge, 2002; Hill et al., 2003), while beta diversity was assessed by principal coordinates analysis (PCoA) based on unweighted UniFrac distances in R. Differences between groups were analyzed using the Wilcoxon test. Linear discriminant analysis effect size (LEfSe) was used to identify differentially abundant taxa. Functional prediction was performed using PICRUSt after normalization for 16S rRNA gene copy number, and predicted functions were annotated against the Kyoto Encyclopedia of Genes and Genomes (KEGG) database to identify enriched metabolic pathways.

### 2.8 Cytokine levels were measured using enzyme-linked immunosorbent assay (ELISA)

Serum levels of insulin, IFN-γ, IL-17, Granzyme B, Perforin 1, TNF-α, IL-1β, IL-6, IL-12, IL-18, IL-10, TGF-β1, and MCP-1, as well as their concentrations in cultured cell supernatants, were quantified using ELISA kits (Bioswamp, Wuhan, China) according to the manufacturer’s instructions. Serum was obtained by centrifugation of coagulated whole blood and stored at −80 °C until analysis. Cell culture supernatants were collected after centrifugation at 3,000 × g for 10 min.

Briefly, samples and biotinylated detection antibodies were added to antibody-coated microplates, followed by horseradish peroxidase-conjugated reagent incubation at 37 °C for 30 min. After washing, chromogenic substrate was added, and the reaction was terminated with stop solution. Optical density was measured at 450 nm using a microplate reader (Bio-Rad, USA), and protein concentrations were determined using standard curves. All assays were performed in triplicate.

### 2.9 Fecal microbiota transplantation (FMT)

To investigate the effect of gut microbiota derived from Omega-3 PUFAs, a FMT experiment was conducted following an established protocol. Prior to transplantation, recipient mice were orally administered antibiotics cocktail (1 g/L ampicillin, 1 g/L neomycin, 1 g/L metronidazole, 0.5 g/L vancomycin Macklin, Shanghai, China) for 7 consecutive days. This was followed by a three-day washout period before FMT (Barcena et al., 2019). Fresh feces were collected daily from NOD mice provided with either a standard diet or an Omega-3 diet. To collect fecal pellets, the abdomens of donor mice were gently palpated to induce reproducible discharge into sterile tubes. Approximately 80–100 mg (four to five fecal pellets) of fresh feces was homogenized in 600 μL of phosphate-buffered saline (PBS) and centrifuged at 500 *g* for 60 s. Then, the recipient mice were intragastrically administered with 150 μL of the supernatant for 7 days. After FMT, the recipient mice were rested for 7 days to allow the transplanted fecal microbiota to colonize before fecal sample collection.

### 2.10 Flow cytometry

For splenic single-cell suspensions separation, mice spleens were gently filtered over 70 μm filter. Then erythrocyte lysis by red blood cell lysis buffer (Beyotime Biotechnology, Shanghai, China), single-cell suspensions were obtained. Surface staining for flow cytometry was performed as previously described (Bi et al., 2017). Briefly, approximately 1 × 10^6^ cells per condition were stained with the following antibodies: FITC-conjugated anti-CD3, alexa fluor 700-conjugated anti-CD4, alexa fluor 700-conjugated anti-CD8a and BV421-conjugated anti-CD25. For intracellular staining, the cells were treated with a permeabilization buffer using BD Cytofix/Cytoperm kit and stained with BV421-conjugated anti-IFN-γ, BV421-conjugated anti-RORγt, PE-conjugated anti-IL-4, PE-conjugated anti-IL-17A, PE-conjugated anti-Foxp3 (all from BD Biosciences) and PE-conjugated anti-H-2Kd InsB Tetramer-LYLVCGERL (Creative Biosciences, Guangzhou, China).

For pancreas single-cell suspensions separation, mice Pancreas were digested with collagenase P and filtered over 70 μm filter. Single-cell suspensions were prepared after lysing erythrocytes. Approximately 1 × 10^6^ cells per condition were stained with the following antibodies: FITC-conjugated anti-CD3, alexa fluor 700-conjugated anti-CD8a and FITC-conjugated anti-CD80 (BioLegend, USA). For intracellular staining, the BD Cytofix/Cytoperm kit was used to permeabilize cells and labeled with PE-conjugated anti-H-2Kd InsB Tetramer-LYLVCGERL.

The cells were examined using flow cytometry. Cells were harvested by trypsinization (Gibco, USA), followed by the addition of media and centrifugation at 500 *g* for 3 mins. Approximately 1 × 10^6^ cells per condition were suspended and stained with FITC-conjugated anti-CD80. For intracellular staining, the cells were permeabilized using BD Cytofix/Cytoperm kit and stained with PE-conjugated anti-CD206 (BioLegend, USA).

Flow cytometry data were collected and analyzed on a CytoFLEX LX instrument (Beckman Coulter) and Flowjo software.

### 2.11 Immunofluorescence (IF) and immunohistochemistry (IHC)

IF and diaminobenzidine (DAB)-based IHC were performed using standard protocols. Paraffin-embedded sections were deparaffinized, rehydrated, treated with 3% hydrogen peroxide to block endogenous peroxidase activity, and subjected to antigen retrieval using citrate buffer (Boster, Wuhan, China). After blocking with 5% BSA, sections were incubated with primary antibodies overnight at 4 °C.

For pancreatic IF staining, sections were incubated with two primary antibodies (mouse anti-insulin antibody, 1:500, 8138S, Cell Signaling Technology and rabbit anti-glucagon antibody, 1:500, SAB4501137, Sigma), followed by two secondary antibodies (alexa fluor 594-conjugated donkey anti-rabbit IgG, 1:200, ab150076, Abcam and alexa fluor 488-conjugated donkey anti-mouse IgG, 1:200, ab150105, Abcam). Nuclei were counterstained with DAPI (ab104139, Abcam), and images were acquired using a laser confocal microscope (Zeiss, Germany).

For IHC staining, sections were incubated with antibodies against CD8 or CD206 (1:100, Servicebio, Wuhan, China), followed by horseradish peroxidase-conjugated secondary antibodies and DAB visualization. Hematoxylin was used for counterstaining. Slides were scanned using a Pannoramic Flash P250 Scanner (3DHistech, Hungary), and quantitative analysis was performed using ImageJ software with the IHC Profiler plugin.

For cellular IF staining, cells were fixed with 4% paraformaldehyde (Biosharp, China), permeabilized with 0.25% Triton X-100 (Biosharp), and blocked with 5% BSA. Samples were incubated overnight at 4 °C with an anti-insulin primary antibody (1:50, Cell Signaling Technology), followed by Alexa Fluor 488-conjugated secondary antibody (1: 400, Abcam) incubation at room temperature. After DAPI counterstaining, images were captured using a fluorescence microscope (Keyence, Japan).

### 2.12 Widely targeted metabolomics analysis of serum

The widely targeted metabolomics analysis of serum was performed by Metware (Wuhan, China). Briefly, samples were stored at −80 °C and then thawed on ice, before being vortexed for 10 s. A 50 μL sample aliquot was combined with 300 μL of an extraction solution (ACN:Methanol, 1:4, V/V) containing internal standards. The mixture was vortexed for 3 mins and centrifuged at 12,000 rpm for 10 mins at 4 °C. A 200 μL supernatant was collected and kept at −20 °C for 30 mins before being centrifuged again at 12,000 rpm for 3 mins at 4 °C. Finally, 180 μL of the clarified supernatant was transferred for LC-MS analysis.

Sample extracts were analyzed using an LC-ESI-MS/MS system (UPLC: ExionLC AD; MS: QTRAP System). Analytical conditions: Waters ACQUITY UPLC HSS T3 C18 column (1.8 µm, 2.1 mm × 100 mm), 40 °C, 0.4 mL/min flow rate, and 2 μL injection. The solvent system was water (0.1% formic acid) and acetonitrile (0.1% formic acid) with a gradient: 95:5 V/V at 0 min, 10:90 V/V at 11.0 min, 10:90 V/V at 12.0 min, 95:5 V/V at 12.1 min, and 95:5 V/V at 14.0 min.

Mass spectrometry was conducted on a QTRAP LC-MS/MS system with an ESI Turbo Ion-Spray interface, using both positive and negative ion modes. Key parameters: source temperature 500 °C, ion spray voltage 5500 V (positive) and −4500 V (negative), gas pressures at 55 psi (GSI), 60 psi (GSII), and 25 psi (CUR). Tuning and calibration used polypropylene glycol solutions, and MRM transitions were monitored for each metabolite.

### 2.13 LS/MS analysis of 18β-GA

Bacterial culture supernatants were snap-frozen in liquid nitrogen for 2 min, thawed and sonicated on ice bath (3 × 5 min), and centrifuged at 15,000 × g for 20 min at 4 °C. 300 μL of the supernatant was mixed with 600 µL methanol. After vacuum-dried, and reconstituted. A 120 µL aliquot was transferred into LC–MS vials. Metabolomics solution was eluted from an ACQUITY UPLC HSS T3 analytical column 1.0 × 50 mm, 1.8 μm with a VanGuard™ Pre-Column using a 10 min gradient from 15% to 98% B with a 10 min total run. The flow rate was 0.17 mL/min, the column oven temperature was maintained at 42 °C. Mobile phase solvents consisted of 66.7% acetonitrile and 0.2 mM ammonium fluoride (A phase) and 10% acetonitrile, 90% isopropanol and 0.2 mM ammonium fluoride (B phase). Mass spectrometry data were acquired using the Data-Dependent Acquisition (DDA) mode. The MS1 full scan covered m/z 150–2000 at a resolution of 30,000, followed by MS/MS scan at 15,000. Positive and negative ion data were acquired using an H-ESI source at + 3500 V and −3000V spray voltages, respectively.

The peak areas of metabolite 18β-GA were normalized to internal standard and averaged within each group. The mean value of the CMC broth (blank) was subtracted from each sample, and the resulting data were log_10_-transformed prior to statistical analysis.

### 2.14 Islet isolation from NOD mice

A solution of collagenase P (1 mg/mL, Roche) in Hank’s Balanced Salt Solution (HBSS, Gibco) was prepared for pancreas digestion. After anesthesia with isoflurane, the mice were sacrificed, a midline incision was made on the abdomen to expose the internal organs, and the pancreas was located near the stomach and spleen. The bile duct was then clipped, and 2 mL of the collagenase P solution was injected into the pancreas tissue. Once the pancreas was filled, it was excised using sterile scissors and transferred into a 15 mL tube containing 2 mL collagenase P solution. The pancreas in the collagenase P solution was incubated at 37 °C for 8 mins in a shaking water bath. The enzymatic reaction was stopped by adding 5 mL of cold HBSS, and the tube was periodically vortexed to ensure even digestion. After centrifuging the digested tissue at 300 g for 3 min, the supernatant was removed, and the islets was isolated by Histopaque-1077 (Sigma) density gradient centrifugation. Under a dissection microscope, the islets were manually picked using a pipette. After isolation, the islets were washed 2-3 times with cold HBSS at 300 g for 3 mins. The supernatant was discarded, and QRIzol (QIAGEN, Germany) was added. The samples were then immediately frozen at −80 °C.

### 2.15 Transcriptomic analysis of islets

Total RNA from pancreatic islets was extracted using QRIzol reagent, and 1 μg was used for library preparation with the VAHTS® Universal V8 RNA-seq Library Prep Kit. Poly(A) mRNA was isolated, fragmented, and converted to cDNA. The cDNA was end-repaired, dA-tailed, and adaptor-ligated, followed by PCR amplification. Indexed libraries were multiplexed and sequenced on a Navoseq6000 platform. Low-quality reads and contaminants were filtered to obtain clean reads for analysis. We used fastp (Chen et al., 2018) for filtering and HISAT (Kim et al., 2015) for mapping. HISAT2 is a faster, sensitive, and highly accurate alignment tool. Clean reads were mapped to the reference gemome using Bowtie2 (Langmead and Salzberg, 2012), and gene expression levels were calculated with RSEM (Li and Dewey, 2011). RSEM is a software tool for estimating gene and isoform expression from RNA-Seq data. Pearson correlation coefficients were calculated to assess sample similarity. Hierarchical clustering was performed with the hclust function in R, and principal component analysis (PCA) was conducted using the princomp function. Data visualization was done using the ggplot2 package in R. Different expression genes (DEGs) were identified using DEGseq and PossionDis, with parameters set to Fold Change ≥1.5 and Adjusted Pvalue ≤0.05. DEGseq (Wang et al., 2010) is based on the Poisson distribution. DEGs were classified based on GO and KEGG annotation results, and functional enrichment analysis was performed using the phyper function in R. The false discovery rate (FDR) was calculated for each term, with terms having an FDR ≤0.05 considered significantly enriched.

### 2.16 Integrative analysis of metabolomics and transcriptomics

Metabolomics and transcriptomics data were processed to identify differentially expressed metabolites (DEMs) and genes (DEGs). The DEMs and DEGs were mapped to the KEGG database and integrated using the joint pathway analysis module in MetaboAnalyst (www.metaboanalyst.ca) (Pang et al., 2024). Enriched pathways were identified based on significance (*P* < 0.05) and impact scores. Visualization tools, including heatmaps, bubble plots, and pathway diagrams, were employed to illustrate the interactions between metabolic and transcriptional changes.

### 2.17 Quantitative real-time polymerase chain reaction (PCR) analysis

Total RNA was extracted using an RNA extraction kit (Tiangen, Beijing, China) according to the manufacturer’s instructions. RNA quality and concentration were assessed with a NanoDrop spectrophotometer (Thermo Fisher, USA), showing A260/A280 ratios around 2.0, indicating pure RNA. A total of 1 µg RNA was reverse transcribed into cDNA using a reverse transcription kit (Vazyme, Nanjing, China) under the conditions: 50 °C for 5 min and 85 °C for 5 s mRNA expression levels of Nos2 and Mrc1 were quantified by real-time PCR using 2× Universal SYBR Green Fast qPCR Mix (Vazyme, Q711), with GAPDH as the internal control. Expression levels were calculated using the 2^(−ΔΔCt)^ method. Primer sequences are provided in Table 1.

**TABLE 1:** Sequence of primers.

Genes	Forward primer (5’→3′)	Reverse primer (5’→3′)
Nos2	GTT​CTC​AGC​CCA​ACA​ATA​CAA​GA	GTG​GAC​GGG​TCG​ATG​TCA​C
Mrc1	TTC​AGC​TAT​TGG​ACG​CGA​GG	GAA​TCT​GAC​ACC​CAG​CGG​AA
Gapdh	AGG​TCG​GTG​TGA​ACG​GAT​TTG	GGG​GTC​GTT​GAT​GGC​AAC​A

### 2.18 Statistical analysis

Data are presented as mean ± standard deviation (SD). For comparisons between two groups, Student’s t-test was used for data following an approximately normal distribution, whereas the Mann–Whitney U test was applied for non-normally distributed data. One-way analysis of variance (ANOVA) was perfomed for comparisons among multiple groups, followed by Bonferroni *post hoc* correction where appropriate. Correlation among gut microbiota, serum cytokines and metabolites were determined using Spearman’s or Pearson’s correlation analysis, depending on data distribution. A two-sided *P* < 0.05 was considered statistically significant. Data analysis was analyzed using GraphPad Prism 9.0 (GraphPad Software).

## 3 Results

### 3.1 Omega-3 PUFA intervention and FMT are associated with altered gut microbiota composition and reduced islet inflammation

**FIGURE 1:**
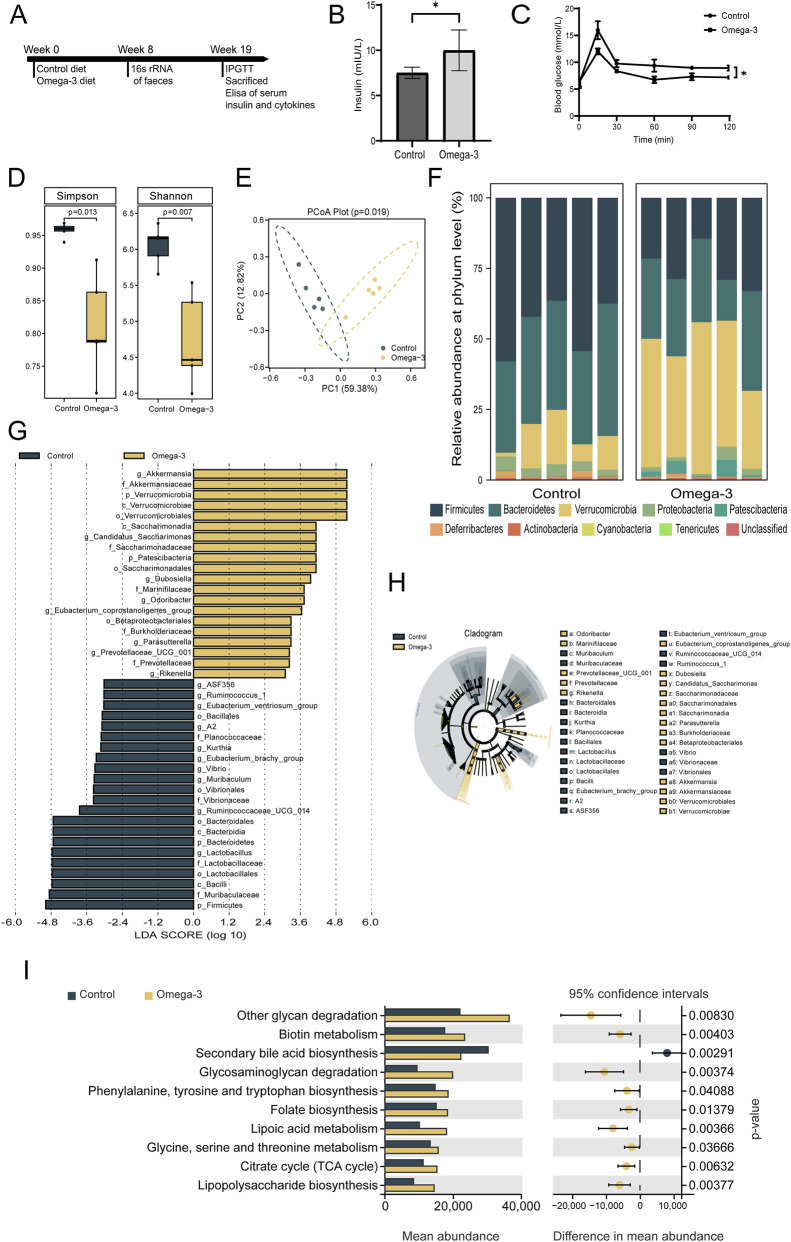
Omega-3 PUFAs altered gut microbiota in NOD mice. **(A)** The process of Omega-3 PUFAs intervention. **(B)** Levels of fast insulin in serum (n = 5). **(C)** Glucose curves of IPGTT (n = 5) **(D)** Alpha diversity box plot (Simpson and Shannon index) in each group of mice (n = 5). **(E)** Principal coordinates analysis (PCoA) using Bray-Curtis of beta diversity. **(F)** Relative abundance of gut microbiota at phylum level. **(G,H)** Taxonomic Cladogram from LEfSe, depicting taxonomic association between microbiome communities from mice. LDA score computed features differentially abundant between the control and Omega-3 mice. The criteria for feature selection are Log(10) LDA Score >2.5. **(I)** PICRUSt functional prediction based on the KEGG orthologs analysis of 16S rRNA sequencing data. Data were analyzed by the Student’s t-test. *P < 0.05, **P < 0.01, ***P < 0.001, ****P < 0.0001, ns: not significant.

We first evaluated the beneficial effects of Omega-3 PUFAs diet on T1DM in NOD mice, and observed significant alterations in gut microbiota composition by 16s rRNA sequencing (Figure 1). To investigate whether Omega-3 PUFA dietary intervention alters gut microbiota composition in NOD mice, 16S rRNA sequencing was performed on fecal samples collected from the Control and Omega-3 groups (n = 5). Alpha diversity analysis based on the Simpson and Shannon indices showed significant differences in microbial diversity between the two groups (Figure 1D). Meanwhile, beta diversity analysis using PCoA revealed a clear separation between the Control and Omega-3 groups, indicating distinct microbial community structures following Omega-3 diet intervention (Figure 1E). At the phylum level, the gut microbiota was dominated by *Firmicutes*, *Bacteroidetes* and *Verrucomicrobia* (Figure 1F). LEfSe analysis further identified several taxa that were significantly enriched in the Omega-3 group (LDA score >2.5), including *Akkermansia, Candidatus saccharimonas, Dubosiella, Odoribacter* and *E. coprostanoligenes* (Figures 1G,H). Functional prediction of the microbial community was performed using PICRUSt and mapped to the KEGG database. Key functional pathways, including other glycan degradation, biotin metabolism and glycosaminoglycan degradation were significantly upregulated in the Omega-3 diet group (P < 0.05) (Figure 1I). Omega-3 PUFAs diet ameliorates insulitis and alters fecal metabolomic profiles in NOD mice. Spearman correlation analysis showed an association between gut microbiota composition and serum cytokine levels (Supplementary Figure S1).

**FIGURE 2:**
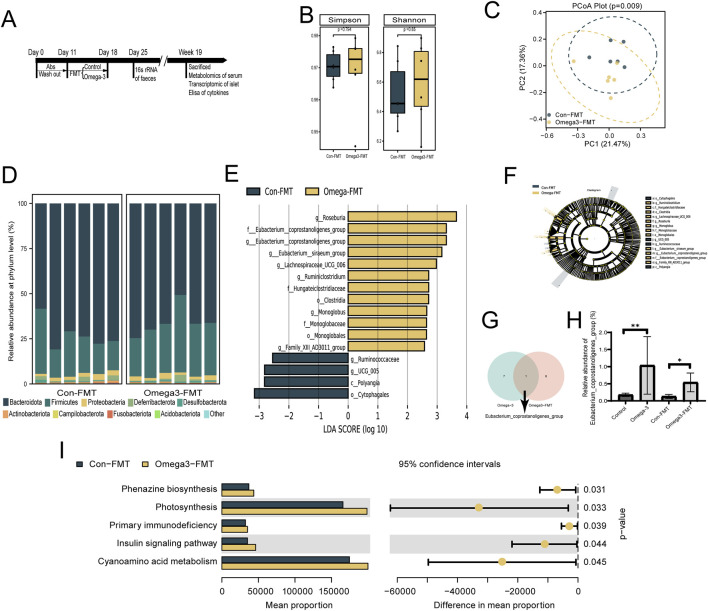
Omega-3 PUFAs-derived gut microbiota altered the gut microbiome in NOD mice. **(A)** The process of FMT intervention. **(B)** Alpha diversity box plot (Simpson and Shannon index) for each group of mice (n = 6). **(C)** Principal coordinates analysis (PCoA) of beta diversity using the Bray-Curtis distance metric. **(D)** Relative abundance of gut microbiota at phylum level. **(E,F)** Taxonomic Cladogram from LEfSe analysis, showing the taxonomic association between microbiome communities in mice. LDA scores were computed to identify features differentially abundant between the Con-FMT and Omega3-FMT mice. The criteria for feature selection were a Log(10) LDA Score >2.5. **(G)** Venn diagram illustrates the overlapped genus *Eubacterium coprostanoligenes group* between the Omega-3 and Omega3-FMT mice. **(H)** Relative abundance of *Eubacterium coprostanoligenes group* in each group. **(I)** PICRUSt functional predictions based on the KEGG ortholog analysis of 16S rRNA sequencing data. Data were analyzed by Mann-Whitney U test. *P < 0.05, **P < 0.01, ***P < 0.001, ****P < 0.0001, ns: not significant.

To further explore the contribution of gut microbiota, FMT was performed to compare immune and inflammatory features between mice receiving Omega-3 PUFAs intervention alone and those receiving microbiota derived from Omega-3 PUFAs-treated donors (Figure 2A; Supplementary Figure S2). Although the Omega3-FMT group exhibited a slightly higher overall incidence of insulitis, the proportion of mice with severe insulitis was lower compared to the Omega-3 group (Supplementary Figure S2a), suggesting a potential modulation of inflammation severity by the microbiota. We next analyzed the levels of cytokines in the serum. Levels of IL-18, IL-6, IL-12, Granzyme B and TNF-α were significantly lower in the Omega3-FMT group compared to the Omega-3 group (Supplementary Figure S2B-F), whereas IFN-γ, IL-17, Perforin-1, and IL-1β showed no apparent differences between the two groups (Supplementary Figure S2G-J). These findings indicate that Omega-3 PUFAs intervention and Omega3-FMT are associated with partially overlapping but distinct immune profiles, suggesting that the gut microbiota may contribute to Omega-3 PUFAs-associated immunomodulatory effects.

To assess microbial engraftment following FMT, 16S rRNA sequencing was performed on fecal samples collected from 12 mice (n = 6). Alpha diversity, measured by Simpson and Shannon indices, showed no notable difference in microbial diversity between the Omega3-FMT group and the Con-FMT group (Figure 2B). Beta diversity, evaluated by using PCoA, revealed differences in the overall community structure (Figure 2C). At the phylum level, the most abundant taxa were *Bacteroidetes*, *Firmicutes* and *Proteobacteria*, with no statistically significant differences observed between the Omega3-FMT and Con-FMT groups (Figure 2D). LEfSe analysis identified seven genera significantly enriched in the Omega3-FMT group (LDA Score >2.5), including *Roseburia*, *E. coprostanoligenes E. siraeum group*, Lachnospiraceae *UCG 006*, *Ruminiclostridiium*, *Monoglobus*, *Family XIII AD3011 group* (Figures 2E,F). Notably, the *E. coprostanoligenes* was enriched in both Omega-3 group and the Omega3-FMT groups (Figure 2G). *Eubacterium coprostanoligenes* could promote goblet cell mucin secretion and enhance the intestinal mucus barrier, which alleviates intestinal mucositis (Bai et al., 2024). The relative abundance was significantly increased in both the Omega-3 group (compared to the control group, *P* < 0.01) and the Omega3-FMT group (compared to the Con-FMT group, *P* < 0.05) (Figure 2H). These results suggest that *E. coprostanoligenes* may play a key role in mediating the protective effects of Omega-3 PUFAs. Functional prediction of the microbial community was performed using PICRUSt and mapped to the KEGG database. Key functional pathways, including phenazine biosynthesis, primary immunodeficiency, and the insulin signaling pathway, were significantly upregulated in the Omega3-FMT group (*P* < 0.05) (Figure 2I).

**FIGURE 3:**
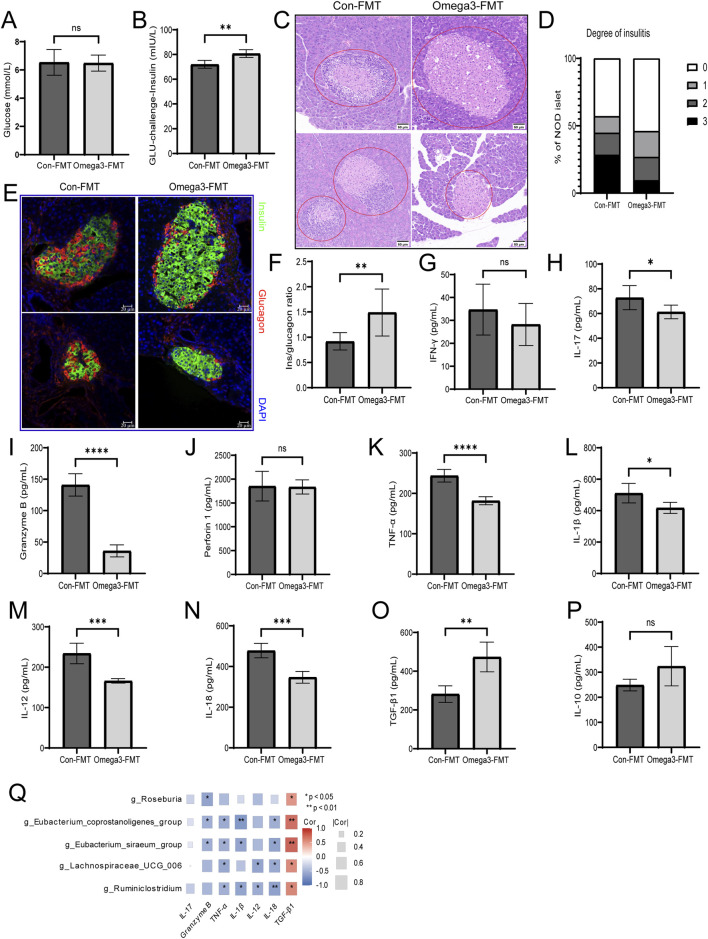
Omega-3 PUFAs-derived gut microbiota protected against the onset of T1DM. **(A)** Serum glucose levels (n = 6). **(B)** Serum insulin levels after glucose challenge (n = 5). **(C)** Representative sections of pancreatic tissue stained with H&E (original magnification ×20) (n = 3). **(D)** Quantification of insulitis incidence in diabetic NOD mice. **(E,F)** Immunofluorescence staining and area ratio of insulin and glucagon in islets for each group. **(G–P)** Cytokine levels measured in serum using their respective ELISA kits. **(G)** IFN-γ. **(H)** IL-17. **(I)** Granzyme B. **(J)** perforin 1. **(K)** TNF-α. **(L)** IL-1β. **(M)** IL-12. **(N)** IL-18. **(O)** TGF-β1 and. **(P)** IL-10 (n = 5). **(Q)** Spearman’s correlation analysis between the top 5 enriched genera in the Omega3-FMT group and differential cytokines based on LEfSe taxonomic analysis. Data were analyzed by the Student’s t-test. *P < 0.05, **P < 0.01, ***P < 0.001, ****P < 0.0001, ns: not significant.

To investigate the potential association between Omega3-FMT and alterations in islet inflammatory and immune features in NOD mice, we monitored NOD mice during the observation period, and no mice developed T1DM (defined as non-fasting blood glucose levels >11.11 mmol/L for two consecutive weeks (Bi et al., 2017)). Mice in the Con-FMT and Omega3-FMT groups showed no statistical differences in random serum glucose (Con-FMT: 6.53 ± 0.91 mmol/L; Omega3-FMT: 6.48 ± 0.56 mmol/L) (Figure 3A) and or fasting insulin levels (not shown). However, following a glucose challenge, the Omega3-FMT groups exhibited significantly higher serum insulin levels compared to the Con-FMT group (Con-FMT: 72.04 ± 3.15 mIU/L; Omega3-FMT: 80.72 ± 3.12 mIU/L; *P* < 0.05) (Figure 3B). Histological assessment of pancreatic sections revealed lower insulitis scores in the Omega3-FMT group (Figures 3C,D). IF microscopy of pancreatic tissue further showed an increased insulin-to-glucagon staining area ratio in the Omega3-FMT group (Figures 3E,F), suggesting a potential protective effects on pancreatic β-cells volume and functions of within the islets of NOD mice.

Serum analysis demonstrated lower levels of several pro-inflammatory cytokines, including IL-17 (Con-FMT: 72.90 ± 9.75 pg/mL; Omega3-FMT: 61.30 ± 5.47 pg/mL), granzyme B (Con-FMT: 141.00 ± 17.82 pg/mL; Omega3-FMT: 36.00 ± 9.62 pg/mL), TNF-α (Con-FMT: 243.75 ± 15.69 pg/mL; Omega3-FMT: 181.88 ± 10.01 pg/mL), IL-1β (Con-FMT: 511.50 ± 62.16 pg/mL; Omega3-FMT: 417.75 ± 35.24 pg/mL), IL-12 (Con-FMT: 234.00 ± 25.30 pg/mL; Omega3-FMT: 166.11 ± 5.52 pg/mL), and IL-18 (Con-FMT: 478.15 ± 35.43 pg/mL; Omega3-FMT: 347.08 ± 28.76 pg/mL), in the Omega3-FMT group compared with the Con-FMT group (*P* < 0.05) (Figures 3H,I,K–N). In contrast, IFN-γ (Con-FMT: 34.71 ± 11.12 pg/mL; Omega3-FMT: 28.24 ± 9.16 pg/mL) and perforin-1 (Con-FMT: 1853.00 ± 311.31 pg/mL; Omega3-FMT: 1836.33 ± 147.19 pg/mL) levels did not differ notably between groups (*P* > 0.05) (Figures 3G,J). The anti-inflammatory cytokine TGF-β1 (Con-FMT: 282.00 ± 42.60 pg/mL; Omega3-FMT: 473.33 ± 76.63 pg/mL) was higher in the Omega3-FMT group (*P* < 0.05) (Figure 3O), whereas IL-10 (Con-FMT: 249.00 ± 23.33 pg/mL; Omega3-FMT: 324.00 ± 78.40 pg/mL) levels were similar between groups (*P* > 0.05) (Figure 3P).

Spearman correlation analysis of gut microbiota composition and serum cytokine levels showed that *E. coprostanoligenes* inversely associated with pro-inflammatory cytokines such as granzyme B (*ρ* = −0.644), TNF-α (*ρ* = −0.681), IL-1β (*ρ* = −0.851), IL-18 (*ρ* = −0.661) and positively associated with anti-inflammatory TGF-β1 (*ρ* = 0.842) (*P* < 0.05) (Figure 3Q). Several additional genera, such as *Eubacterium siraeum group*, Lachnospiraceae *UGG 006*, and *Ruminiclostridium*, were also shown to have inverse associations with pro-inflammatory cytokines (Figure 3Q), although enrichment across experimental groups was most consistent for the *E. coprostanoligenes*.

### 3.2 Omega3-FMT is associated with altered macrophage polarization in the islet microenvironment

**FIGURE 4:**
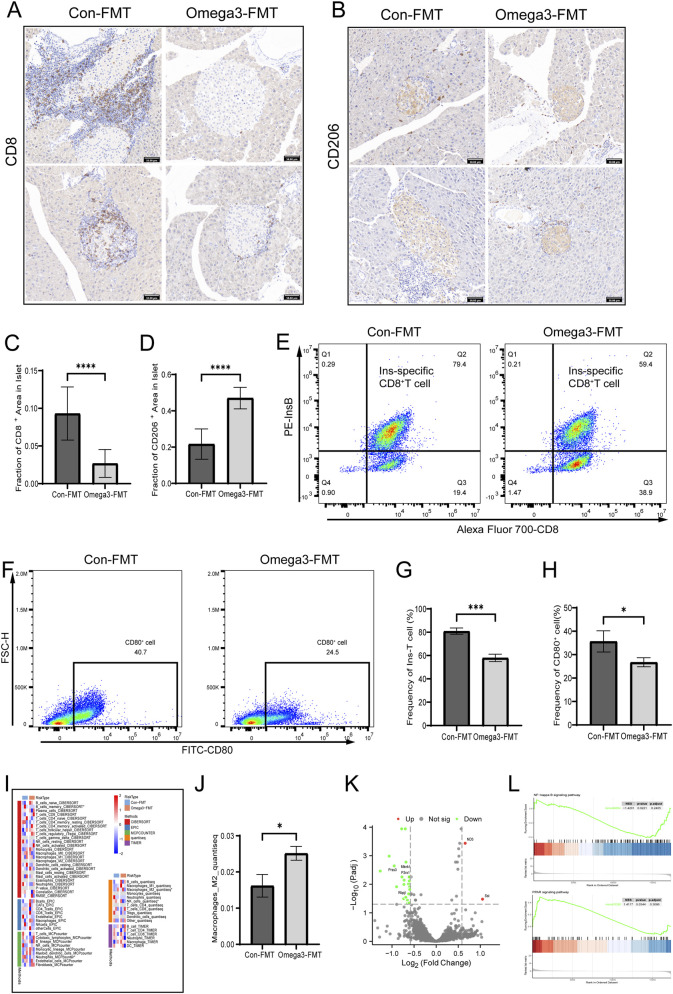
Omega-3 PUFAs-derived gut microbiota reduced M1 macrophages proportion in the islet microenvironment. **(A,C)** Immunohistochemistry and quantification of CD8 in islets of each group (n = 3). **(B,D)** Immunohistochemistry and quantification of CD206 in islets of each group (n = 3). **(E,G)** Flow cytometric image and percentages of insulin-specific CD8^+^ T Cell in pancreas (n = 3). **(F,H)** Flow cytometric image and percentage of CD80^+^ cell in the pancreas (n = 3). **(I)** Immune infiltrate analyzes. **(J)** Proportion of M2 macrophages in quantiseq algorithm. **(K)** Differential genes expressed between the Con-FMT and Omega3-FMT group (n = 3). **(L)** Gene set enrichment analysis. Data were analyzed by the Student’s t-test. *P < 0.05, **P < 0.01, ***P < 0.001, ****P < 0.0001, ns: not significant.

Immunohistochemical analysis revealed lower CD8 expression and higher CD206 expression in pancreatic islets from the Omega3-FMT group compared with the Con-FMT group (Figures 4A–D). Flow cytometry analysis also showed a reduced proportion of insulin-specific CD8^+^ T Cells (80.90% ± 2.69% vs. 57.93% ± 3.17%, *P* < 0.05) and CD80^+^ cells (Con-FMT: 35.63% ± 4.55%; Omega3-FMT: 26.73% ± 1.94%, *P* < 0.05) in the pancreas of the Omega3-FMT group (Figures 4E–H). Nevertheless, no marked difference was observed in the proportion of splenic Th1, Th2, Rorγt^+^ Th17, and Foxp3^+^ Treg cells between the two groups (Supplementary Figures S23A-D). These results suggested that Omega-3 PUFAs may exert immune regulatory effects mainly on CD8^+^ T Cell or M2 macrophages but not CD4^+^ T Cells through gut microbiota shift.

Further, transcriptomic immune cell deconvolution using the quantiseq algorithm indicated a higher proportion of M2 macrophages in the islets from Omega3-FMT group (Figures 4I,J). Differential gene expression analysis of islet tissue revealed downregulation of several inflammation-associated genes (Prss3, Mknk1, P2rx1, Rbpjl), while gene set enrichment analysis showed a trend toward suppression of NF-κB signaling and enrichment of PPAR pathways, although statistical significance was attenuated after multiple-testing correction (Figures 4K,L). These findings suggest an association between Omega3-FMT and a shift toward an anti-inflammatory macrophage phenotype in the islet microenvironment, potentially contributing to the protective effects on islet function.

### 3.3 Omega3-FMT is associated with increased serum levels of 18β-GA

**FIGURE 5:**
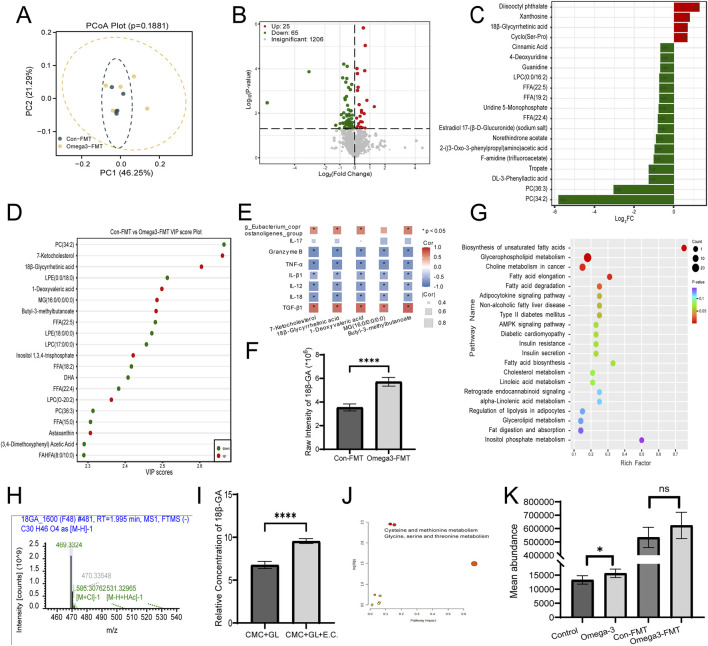
Omega-3 PUFAs-derived gut microbiota elevated levels of 18β-GA in serum. **(A)** PCoA based on Bray-Curtis of serum metabolomics analysis (n = 5). **(B)** Volcanic map showing differential metabolites between the Con-FMT and Omega3-FMT group. **(C)** Bar chart depicting the top 20 differential metabolites between the groups. **(D)** The top 20 metabolites with Variable importance in projection (VIP) scores. **(E)** Pearson’s correlation analysis between the top 5 upregulation metabolites (based on VIP scores), the relative abundance of the *Eubacterium coprostanoligenes group*, and differential cytokines. **(F)** Raw intensity of 18β-GA. **(G)** KEGG pathway enrichment analysis of differential metabolites. **(H)** Extracted ion chromatogram (EIC) of 18β-GA detected in ESI mode, showing a precursor ion of m/z 469.3324. **(I)** Relative concentration of 18β-GA in bacterial culture supernatants. **(J)** Integrative analysis of serum metabolomics and islet transcriptomics. **(K)** Mean abundance of the glycine, serine, and threonine metabolism pathway predicted by PICRUSt based on fecal 16S rRNA sequencing for each group. Data were analyzed by the Student’s t-test. *P < 0.05, **P < 0.01, ***P < 0.001, ****P < 0.0001, ns: not significant.

To investigate metabolic alterations linked to Omega3-FMT, serum metabolomics profiling was performed. PCoA analysis did not reveal a significant separation between the groups at the overall metabolic level (Figure 5A). However, differential metabolomics analysis identified 25 metabolites with increased levels and 65 metabolites with decreased levels relative to the Con-FMT group (Figure 5B).

A bar chart highlighting the top 10 differentially expressed metabolites by fold change identified 18β-GA as one of the key metabolites (Figure 5C). Variable importance in projection (VIP) scores from the PLS-DA analysis further supported the importance of 18β-GA, as it ranked among the top-ranking metabolites (Figure 5D).

Pearson correlation analysis was performed between the top 5 upregulated metabolites identified by the VIP scores, the abundance of the *E. coprostanoligenes*, and levels of serum cytokines. The analysis revealed that these metabolites, including 18β-GA, were positively correlated with the *E. coprostanoligenes* (*r* = 0.748) and TGF-β1 (*r* = 0.778) levels, while negatively correlated with pro-inflammatory cytokines, such as granzyme B (*r* = −0.765), TNF-α (*r* = −0.771), IL-1β (*r* = −0.750), IL-12 (*r* = −0.745) (Figure 5E). Widely targeted metabolics analysis of serum showed higher raw intensity of 18β-GA in the Omega3-FMT group (Con-FMT:3,540,415.9 ± 292,563.99; Omega3-FMT:5,714,729.8 ± 366,357.93, *P* < 0.05).

KEGG pathway enrichment analysis of the differentially expressed metabolites indicated involvement in pathways associated with the biosynthesis of unsaturated fatty acids, glycerophospholipid metabolism, choline metabolism in cancer, fatty acid elongation, and fatty acid degradation. These findings suggest that the observed metabolic changes may play a role in modulating fatty acid metabolism (Figure 5G).

An *in vitro* bacterial culture experiment was performed to investigate the potential involvement of the *E. coprostanoligenes* in metabolic processes associated with 18β-GA generation under experimental conditions, and increased 18β-GA levels were detected by targeted LC-MS/MS analysis under ESI mode (Figure 5H). Relative abundance of 18β-GA in the CMC broth + *E. coprostanoligenes* + GL group was markedly higher than in the CMC broth + GL group (6.130 ± 0.419 VS. 9.121 ± 0.279, *P* < 0.0001) (Figure 5I). These results indicate that the presence of *E. coprostanoligenes* may lead to a significant accumulation of 18β-GA, suggesting its microbial origin.

Integrative analysis of serum metabolomics and islet transcriptomics revealed a significant enrichment of pathways, as shown in the accompanying figure (Figure 5J). The top enriched pathways included cysteine and methionine metabolism (impact scores = 0.13, *P* = 0.0035) and glycine, serine, and threonine metabolism (impact scores = 0.15, *P* = 0.0037). PICRUSt prediction based on fecal 16S rRNA sequencing indicated that the glycine, serine, and threonine metabolism pathway was more enriched in the Omega-3 group compared to the control group (*P* < 0.05). In the Omega3-FMT group, there was a trend towards higher enrichment of the glycine, serine, and threonine metabolism pathway compared to the Con-FMT group, although this difference did not reach statistical significance (Figure 5K). These results suggest that the glycine, serine, and threonine metabolism pathway may play an important regulatory role in the gut-islet axis.

### 3.4 18β-GA is associated with macrophage polarization toward an M2-like phenotype and altered β-cell insulin responses

Previous studies have demonstrated that 18β-GA is involved in the regulation of macrophage polarization. To examine its effects in an inflammatory context, RAW264.7 macrophages were activated with lipopolysaccharide (LPS) to induce a pro-inflammatory M1 phenotype prior to 18β-GA intervention (Tian et al., 2021).

The Nos2 gene encodes inducible nitric oxide synthase (iNOS), a key enzyme involved in nitric oxide production and commonly used as a marker of M1 macrophage activation (Guo et al., 2023; Klug et al., 2013). LPS treatment markedly increased Nos2 expression compared to the negative control (NC) (*P* < 0.0001). While 10 µM 18β-GA had no significant effect, 20 µM 18β-GA significantly reduced Nos2 expression compared to LPS group (*P* < 0.0001) (Supplementary Figure S4A). Similarly, 20 µM 18β-GA significantly increased Mrc1 expression compared to both the LPS group and LPS +10 µM 18β-GA group (*P* < 0.05) (Supplementary Figure S4B).

Flow cytometry analysis showed that LPS treatment increased the mean fluorescence intensity (MFI) of CD80 compared to the NC group (*P* < 0.0001). Treatment with 10 µM 18β-GA and 20 µM 18β-GA was associated with lower CD80 MFI relative to LPS treatment alone (*P* < 0.05) (Supplementary Figures S4C,D). In contrast, CD206 expression was not markedly altered by LPS alone but showed higher MFI following treatment with 20 μM 18β-GA compared with the LPS and LPS +10 μM 18β-GA groups (*P* < 0.05) (Supplementary Figures S4E,F), consistent with a shift toward an M2-like macrophage phenotype.

**FIGURE 6:**
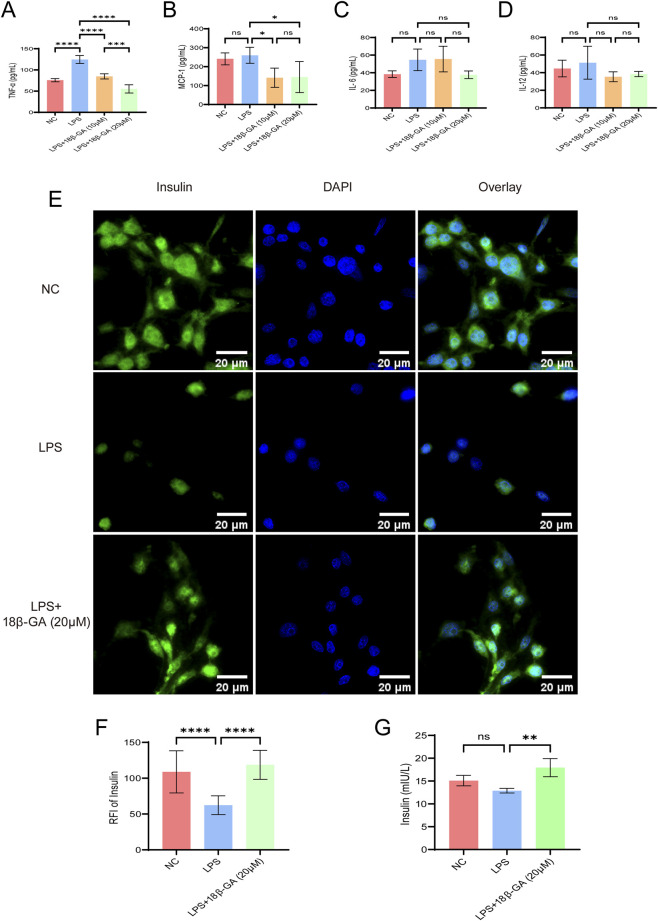
18β-GA protects pancreatic β-cells by promoting M2 macrophage polarization. **(A–D)** Cytokines levels, including. **(A)** TNF-α. **(B)** MCP-1 **(C)** IL-6, and. **(D)** IL-12, in macrophages culture supernatants were measured using their respective ELISA kit (n = 4). **(E,F)** Immunofluorescence staining and qualification of insulin in β cells co-culture with macrophages (n = 3). **(G)** Insulin levels in the supernatant of β cell and macrophage co-cultures (n = 4). Data were analyzed by ordinary one-way ANOVA with Bonferroni correction. *P < 0.05, **P < 0.01, ***P < 0.001, ****P < 0.0001, ns: not significant.

ELISA analysis of cytokine levels in macrophage culture supernatants showed that LPS treatment increased TNF-α levels compared with the NC group (NC: 75.83 ± 3.79 pg/mL vs. LPS: 124.40 ± 9.29 pg/mL, *P* < 0.05). Treatment with 18β-GA at concentrations of 10 µM (84.79 ± 6.06 pg/mL) and 20 µM (55.21 ± 9.78 pg/mL) was associated with a reduction in TNF-α levels in a dose-dependent manner (*P* < 0.05) (Figure 6A). MCP-1 levels in the supernatants were comparable between the NC and LPS groups (NC: 241.10 ± 31.46 pg/mL vs. LPS: 259.60 ± 42.30 pg/mL, *P* > 0.05). In contrast, treatment with 18β-GA at 10 µM (141.40 ± 50.72 pg/mL) and 20 µM (145.00 ± 81.95 pg/mL) was associated with lower MCP-1 levels compared with the LPS group (*P* < 0.05) (Figure 6B). No significant changes were noted in IL-6 and IL-12 levels across all groups (Figures 6C,D).

IF staining for insulin in NIT-1 β-cells co-culture with LPS-activated macrophages revealed a marked decrease in insulin-positive cells compared to the NC co-culture group. In contrast, insulin staining intensity appeared higher in the LPS +20 µM 18β-GA co-culture group relative to the LPS group (Figures 6E,F). Consistent with these observations, insulin concentrations in the culture supernatants were comparable between the NC (15.10 ± 1.14 mIU/L) and LPS (12.89 ± 0.52 mIU/L) co-culture groups, whereas higher insulin levels were detected in the LPS +20 µM 18β-GA co-culture group (17.94 ± 1.99 mIU/L) (Figure 6G). Together, these findings suggest that 18β-GA treatment is associated with attenuation of LPS-induced inflammatory responses in macrophages and preservation of β-cell insulin production in the co-culture system.

## 4 Discussion

T1DM, a chronic autoimmune endocrine disorder, results from a combination of genetic susceptibility factors and environmental factors that trigger an abnormal immune response against the islets of Langerhans (Kobayashi and Kadowaki, 2024). Although a lot of achievements have been made in insulin therapy and glucose monitoring in T1DM patients, T1DM still poses a major health burden with the rising incidence all over the world, especially in young people (Groele and Szypowska, 2023). Therefore, a better understanding of the mechanisms underlying β-cell autoimmunity is urgently needed to identify novel immune-regulatory and microbiota-associated pathways relevant to this disease beyond the therapy provided when symptomatic.

Recent studies have revealed a potential association between Omega-3 PUFAs and immune modulation T1DM (Bi et al., 2017), with gut microbiota increasingly recognized as a potential contributing factor (Lo Conte et al., 2022; Biassoni et al., 2020). However, due to the intricate interaction between gut microbiota, the immune system, and T1DM, the molecular mechanisms linking Omega-3 PUFAs-derived gut microbiota to T1DM remain unclear. Here we describe a potential gut-islet interaction in which both dietary Omega-3 PUFAs and gut microbiota transfer were associated with reduced islet inflammation in a NOD mouse model for T1DM. In the present study, our finding suggests that Omega-3 PUFAs intervention is associated with modulation of gut microbiota, including enrichment of the *E. coprostanoligenes*, which showed a close association with elevated levels of 18β-GA. This elevation was associated with modulation of the gut-pancreas axis, accompanied by changes in the balance of M1 and M2 macrophages in the islet immune microenvironment. The observed shift towards an anti-inflammatory M2 macrophage phenotype may contribute to altered inflammatory features and β-cell function responses.

It has been reported that the relative abundance of *Akkermansia muciniphila* increases significantly in T1DM NOD mice after Omega-3 PUFAs enriched diets (Lo Conte et al., 2022). Our findings revealed an increased abundance of the genus *A. muciniphila* in the Omega-3 PUFAs group compared to the control group. However, no apparent difference in its prevalence was detected between the Con-FMT and Omega3-FMT groups. Several factors may explain the failure of *A. muciniphila* colonization after transplantation. First, the construction of a pseudo-germ-free mouse model using broad-spectrum antibiotics and a short washout period may differentially affect bacterial taxa, as microbial sensitivity to antibiotics varies considerably among species (Lavelle et al., 2019). Studies have shown that certain strains of *A. muciniphila* exhibit heightened sensitivity to antibiotics (Li et al., 2024), whereas others may persist or even expand under specific antibiotic conditions, such as vancomycin exposure (Hansen et al., 2012). Second, *A. muciniphila* relies on mucin as a nutrient source and prefers relatively alkaline environments (Hou et al., 2023), suggesting that gastric acidity during transplantation may limit its survival and engraftment. Despite the lack of sustained *A. muciniphila* enrichment after FMT, recipient NOD mice exhibited reduced insulitis severity and altered immune features. These observations suggest that Omega-3 PUFAs-associated gut microbiota may influence inflammatory and immune characteristics relevant to T1DM through multiple bacterial taxa, rather than dependence on a single microbial species.

The protective effect of gut microbiota against islet inflammation are unlikely be limited to a single genus. Several bacterial taxa showing inverse associations with pro-inflammatory cytokines may collectively contribute to immune regulation. Notably, the *E. coprostanoligenes* was consistently enriched in both the Omega3-FMT group and the Omega-3 PUFAs diet group, suggesting a reproducible association with Omega-3 PUFAs intervention. Supporting this observation, an increased abundance of *Eubacterium* was reported in a healthy adult following short-term Omega-3 PUFAs supplementation (Noriega et al., 2016), indicating that this bacterial genus may respond to Omega-3 PUFAs intake across species. The *E. coprostanoligenes* is recognized for its ability to convert cholesterol into coprostanol, a process that may influence lipid metabolism and immune function (Kim et al., 2022; Shao et al., 2019). Previous studies have suggested that this bacterial group may be involved in modulation of immune responses and inflammatory pathways (Li et al., 2022). In addition, Guo et al. identified that *E. coprostanoligenes* as a microbial feature closely associated with T1DM complications (Guo et al., 2024). Together, these findings highlight the potential relevance of the *E. coprostanoligenes* in gut microbiota-immune-metabolic interactions related to T1DM, while underscoring the contribution of multiple microbial taxa.

In our study, integrated analyzes including transcriptomic profiling of pancreatic islets, flow cytometry, and immunohistochemical staining indicated that Omega3-FMT was associated with changes in macrophage polarization within the islet microenvironment of NOD mice, characterized by increased M2 polarization and reduced M1 polarization. Previous studies have shown that M2 macrophage polarization exerts protective effects against T1DM by mitigating pancreatic inflammation, promoting tissue repair, and enhancing immune regulation (Espinoza-Jimenez et al., 2012). The anti-inflammatory properties of M2 macrophage have been linked to the secretion of cytokines such as IL-10 and TGF-β1, which may suppress pro-inflammatory pathways and limit β-cell damage (Chen et al., 2019; Gong et al., 2012). Increasing evidence suggests that bioactive compounds and microbiota-associated metabolites can modulate macrophage polarization and inflammatory responses, thereby contributing to immune homeostasis in chronic inflammatory diseases (Adhikary et al., 2024b).

Using MHC-I tetramer staining to identify insulin-specific CD8^+^ T Cells, we observed a reduced proportion of these cells within the islet microenvironment of Omega3-FMT mice, accompanied by a relative increase in the spleen. The mechanisms underlying this redistribution remain unclear and warrant further investigation. M1 macrophages produce pro-inflammatory cytokines and chemokines such as TNF-α, IL-1β, and chemokine like CCL2 and CXCL10 (Qin et al., 2012; Sezginer and Unver, 2024), which may influence the recruitment and localization of cytotoxic T Cells. Alterations in macrophage-associated inflammatory signals could therefore affect CD8^+^ T Cell trafficking between peripheral lymphoid organs and pancreatic islets, although this hypothesis remains speculative.

In contrast, no marked differences in CD4^+^ T Cell subsets (Th1, Th17, and Treg) were observed in the Omega3-FMT group, despite the established involvement of these cells in T1DM pathogenesis (Katsarou et al., 2017). Consistent with our previous findings, direct Omega-3 PUFAs supplementation was associated with altered CD4^+^ T Cell differentiation in the spleen (data not shown) (Bi et al., 2017), whereas such changes were not observed following Omega3-FMT. However, the modulation of T Cell subset differentiation might exert adverse effects on anti-infective or anti-tumor immunity (Baldwin et al., 2025; Niehaus et al., 2025). This discrepancy suggests that modulation of the gut microbiota represents only one component of Omega-3 PUFAs-associated immunoregulation, and that additional microbiota-independent mechanisms are likely involved. Meanwhile, the observed modulation of CD8^+^ T Cells and macrophages suggests that the primary immunomodulatory effects of Omega-3 PUFAs-associated microbiota in our model may operate through cytotoxic and innate immune pathways, with less direct impact on CD4^+^ T Cell differentiation (Gill et al., 2022; Fu et al., 2021). By directly comparing the Omega-3 and Omega3-FMT groups, we found FMT reduced the severity of insulitis and decreased levels of multiple cytokines, most of which are primarily derived from macrophages. These findings suggest that gut microbiota may exert immunomodulatory effects mainly by influencing macrophages and possibly CD8^+^ T Cells, with comparatively limited impact on CD4^+^ T Cell subsets, which may contribute to maintaining immune homeostasis while minimizing broad alterations in adaptive immune cell subsets.

Serum metabolite profiling in FMT recipient mice revealed several differential metabolites, including PC (34:2), 7-ketocholesterol, 18β-GA and LPE (0:0/18:0). Among these, 18β-GA exhibited one of the highest change and VIP (Variable Importance in Projection) score, indicating its significant contribution to metabolic differences between groups. Furthermore, 18β-GA correlated positively with the abundance of the *E. coprostanoligenes*. Prior studies have suggested that *Eubacterium* species convert glycyrrhizic acid (GL) into 18β-GA (Kim et al., 2000), and our *in vitro* bacterial culture experiments showed increased 18β-GA levels in the presence of *E. coprostanoligenes* under experimental conditions. 18β-GA exerts protective effects on the immune system through anti-inflammatory and immunomodulatory actions (Stecanella et al., 2021).

In addition to immune-related changes, enrichment of the glycine, serine, and threonine metabolism pathway was observed in the Omega3-FMT group. These amino acid metabolic pathways have been implicated in regulation of inflammatory processes and intestinal function (Das et al., 2021). Threonine is utilized by the gut and plays a critical role in mucin synthesis and maintenance of intestinal barrier integrity (Yang and Liao, 2019). Meanwhile, Omega-3 PUFAs influence the gut microbiota by favoring beneficial and butyrate-producing bacteria while reducing LPS-producing taxa, which could potentially be associated with alterations in inflammatory signaling pathways, including NF-κB-related pathways (Bascunan et al., 2025). Collectively, these observations suggest that Omega-3 PUFAs-associated alterations in gut microbiota and amino acid metabolism composition may be linked to changes in intestinal homeostasis and inflammatory features relevant to T1DM.

Although levels of insulin in the cell culture supernatant did not differ markedly between NC co-culture group and the LPS co-culture group, this does not indicate that LPS-induced M1 macrophages have no damaging effect on pancreatic islet cells. We hypothesized that when β-cells underwent death and lysis, intracellular insulin was released into the culture supernatant, potentially affecting the measured insulin levels in the supernatant. Also, our study suggested that β-cells co-cultured with LPS+18β-GA-treated macrophages exhibited higher insulin levels in the culture supernatant than that co-cultured with LPS-treated macrophage. This observation may reflect altered β-cell responses under inflammatory conditions, although additional studies are required to clarify the underlying mechanisms.

However, this study has certain limitations. First, germ-free mice were not used as FMT recipients, and the administration of broad-spectrum antibiotics could have influenced the composition of the gut microbiota. Second, the analysis of metabolic pathways was based on functional prediction from 16s rRNA sequencing rather than shotgun metagenomic sequencing, which may limit the accuracy. Third, microbiota depletion experiments and *in vivo* 18β-GA supplementation experiments were not performed, and direct causal relations between Omega-3 PUFAs-associated microbiota alterations, 18β-GA and immune modulation, respectively, could not be confirmed. Also, although an association of *E. coprostanoligenes* with an increased level of 18β-GA was demonstrated, the present results cannot exclusively establish direct biosynthesis or production of 18β-GA by that bacterial group. As for transcriptomic pathway analysis, this study was exploratory because statistical significance of both pathways was reduced by correction for multiple-testing, and further targeted validation for NF-κB and PPAR related pathways is needed. Also, the sample size was small and this makes an analytical result such as a multi-omics analysis prone to false positive results. Moreover, this study was also conducted exclusively on the female NOD mouse model. On the basis of known differences of T1DM incidence and gut microbiota between sexes, the generalizability of these results to male subjects should be interpreted cautiously. Also, none of the mice in this study developed overt T1DM during the follow-up period; thus, our results cannot be interpreted as proof to prevent or delay diabetes onset. Furthermore, our current study was performed mainly in NOD mice and *in vitro* systems and may not fully reflect the complexity of T1DM in humans. Finally, we used the NIT-1 pancreatic β-cell line and RAW264.7 macrophage cell line, which, while informative, do not fully replicate the physiological conditions of primary cells. Future studies are needed to further validate these findings and clarify the underlying mechanisms.

In conclusion, our study describes a new gut-islet axis by which changes in gut microbiota due to Omega-3 PUFAs have the potential to modulate inflammation features relevant to T1DM. We identify microbial metabolite 18β-GA as the mediator that promotes M2 macrophage polarization and thereby islet immune suppression and β-cells function. An integrative multi-omics analysis also brings out the potential role of glycine, serine and threonine metabolism in this potential mechanism. Our data moved understanding away from general dietary regimens to microbiota-mediated therapy. These study gives additional insights into microbiota-metabolite-immune interactions relevant to T1DM and warrants further work into the microbiota-associated metabolic pathways in autoimmune diseases.

## Data Availability

The raw 16S rRNA and RNA sequencing data have been deposited in the NCBI Sequence Read Archive (SRA) under BioProject accession number PRJNA1291847, and the metabolomics raw data generated in this study have been stored in Metabolights database under the accession number MTBLS13006 (mice serum), MTBLS13979 (mice feces) and MTBLS13174 (bacterial culture supernatants).

## Glossary

T1DM: Type 1 diabetes mellitus
PUFAs: Polyunsaturated fatty acids
ALA: Alpha-linolenic acid
EPA: Eicosapentaenoic acid
DHA: Docosahexaenoic acid
FMT: Fecal microbiota transplantation
NOD: Non-obese diabetic
E. coprostanoligenes: Eubacterium coprostanoligenes group
18β-GA: 18β-glycyrrhetinic acid
GL: Glycyrrhizic acid
CMC: Chopped Meat Carbohydrate (broth)
SPF: Specific pathogen-free
IPGTT: Intraperitoneal glucose tolerance test
H&E: Hematoxylin and eosin
PCoA: Principal coordinates analysis
LEfSe: Linear discriminant analysis effect size
LDA: Linear discriminant analysis
PICRUSt: Phylogenetic Investigation of Communities by Reconstruction of Unobserved States
KEGG: Kyoto Encyclopedia of Genes and Genomes
ELISA: Enzyme-linked immunosorbent assay
PBS: Phosphate-buffered saline
LPS: Lipopolysaccharide
IF: Immunofluorescence
IHC: Immunohistochemistry
DAB: Diaminobenzidine
BSA: Bovine serum albumin
DAPI: 4′,6-diamidino-2-phenylindole
LC–MS/MS: Liquid chromatography–tandem mass spectrometry
UPLC: Ultra-performance liquid chromatography
ESI: Electrospray ionization
MRM: Multiple reaction monitoring
DDA: Data-dependent acquisition
HBSS: Hank’s Balanced Salt Solution
PCA: Principal component analysis
DEGs: Differentially expressed genes
GO: Gene Ontology
FDR: False discovery rate
PCR: Polymerase chain reaction
SD: Standard deviation
ANOVA: Analysis of variance
MFI: Mean fluorescence intensity
DMEM: Dulbecco’s modified Eagle’s medium
