# Supplementary material for: Omega-3 polyunsaturated fatty acids are associated with microbiota-related 18β-glycyrrhetinic acid alterations and M2 macrophage polarization in type 1 diabetes mellitus
Source: Front Pharmacol. 2026 Jun 17;17:1871892. doi: 10.3389/fphar.2026.1871892 (PMC13318763; doi:10.3389/fphar.2026.1871892)
Supplement: Supplementary file 1 [file Supplementaryfile1.docx]

**Table S1.** Compositions of control diet.

| Basic Diet | Conventional |
| --- | --- |
| Ground corn (8% protein), g/kg | 483 |
| Bran (15% protein), g/kg | 120 |
| Ground wheat (13% protein), g/kg | 170 |
| Soybean meal (44% protein), g/kg | 70 |
| Fish meal (68% protein), g/kg | 40 |
| Chicken meal (68% protein), g/kg | 50 |
| Meal premix (34% protein), g/kg | 46 |
| Ground Limestone, g/kg | 11 |
| Soybean oil, g/kg | 1 |
| Lysine, g/kg | 13.5 |
| Methionine + Cystine, g/kg | 7.6 |
| Arginine, g/kg | 11.9 |
| Histidine, g/kg | 6.0 |
| Tryptophan, g/kg | 3.6 |
| Phenylalanine + Tyrosine, g/kg | 14.9 |
| Threonine, g/kg | 8.7 |
| Leucine, g/kg | 14.5 |
| Isoleucine, g/kg | 9.5 |
| Valine, g/kg | 10.5 |
| Magnesium, g/kg | 2.27 |
| Potassium, g/kg | 6.53 |
| Sodium, g/kg | 3.11 |
| Iron, mg/kg | 195 |
| Manganese, mg/kg | 105 |
| Copper, mg/kg | 14.1 |
| Zinc, mg/kg | 55 |
| Iodine, mg/kg | 0.55 |
| Selenium, mg/kg | 0.12 |
| Vitamin A, IU/kg | 13692 |
| Vitamin D, IU/kg | 1339 |
| Vitamin E, IU/kg | 115.4 |
| Vitamin K, mg/kg | 5.5 |
| Vitamin B1, mg/kg | 15 |
| Vitamin B2, mg/kg | 17.4 |
| Vitamin B6, mg/kg | 10 |
| Vitamin B12, mg/kg | 0.035 |
| Niacin, mg/kg | 74.9 |
| Pantothenic acid, mg/kg | 32.7 |
| Biotin, mg/kg | 0.174 |
| Choline, mg/kg | 1360 |
| Folic acid, mg/kg | 7.5 |

**Figure legend**

**Figure S1.** Omega-3 PUFA diets ameliorate insulitis and alter fecal metabolomics profiles in NOD mice.

(A) Representative sections of pancreas by hematoxylin–eosin (H&E) staining (original magnification × 20) (n=4). (B) Quantification of the incidence of insulitis in diabetic NOD mice. Cytokines including (C) IFN-γ, (D) IL-18, (E) IL-17, (F) perforin 1, (G) IL-1β, (H) IL-6 in serum were measured with their respective ELISA kit (n=5). Data were analyzed by the student's t-test. (I) Spearman’s correlation analysis between the Omega-3 group enriched genus (top 5) and differential cytokines base on the taxonomic analysis of LEfSe. (J) Peak area of representative significantly altered fecal metabolites (adj.P < 0.05) identified by untargeted LC–MS/MS metabolomics in the control diet group and the Omega-3 PUFAs diet group. Metabolite abundance is represented by LC–MS peak areas (Area ×10⁶). Bars represent mean ± SD, and each dot indicates an individual mouse (n = 5). **P* < 0.05, ***P* < 0.01, ****P* < 0.001, *****P* < 0.0001, ns: not significant.

**Figure S2.** The gut microbiota may mediate the immunoregulatory effects of Omega-3 PUFAs. (A) Quantification of the incidence of insulitis in diabetic NOD mice. Cytokines including (B) IL-18, (C) IL-6, (D) IL-12, (E) Granzyme B, (F) TNF-α, (G) IFN-γ, (H) IL-17, (I) perforin 1, (J) IL-1β in serum were measured with their respective ELISA kit (n=5).*P < 0.05, **P < 0.01, ***P < 0.001, ****P < 0.0001, ns: not significant.

**Figure S3.** Omega-3 PUFAs-derived gut microbiota elevated the percentage of insulin-specific CD8^+^ T cells in the spleen. Flow cytometric image and percentage of (A) Th1, (B) Th2, (C) Rorγt^+^ Th17, (D) Foxp3^+^ Treg, (E) insulin-specific CD8^+^ T cell in spleen of NOD mice (n=4). Data were analyzed by the student's t-test. **P* < 0.05, ***P* < 0.01, ****P* < 0.001, *****P* < 0.0001, ns: not significant.

**Figure S4.** 18β-GA promotes M2 macrophage polarization. Quantitative real-time PCR analysis of (A) Nos2 and (B) Mrc1 between each group (n=3). Flow cytometric images and percentage of (C, D) M1 and (E, F) M2 macrophage cells (n=3). Data were analyzed by ordinary one-way ANOVA and Bonferroni correction. **P* < 0.05, ***P* < 0.01 ****P* < 0.001, *****P* < 0.0001.
