# Supplementary figures and images for: Omega-3 polyunsaturated fatty acids are associated with microbiota-related 18β-glycyrrhetinic acid alterations and M2 macrophage polarization in type 1 diabetes mellitus
Source: Front Pharmacol. 2026 Jun 17;17:1871892. doi: 10.3389/fphar.2026.1871892 (PMC13318763; doi:10.3389/fphar.2026.1871892)

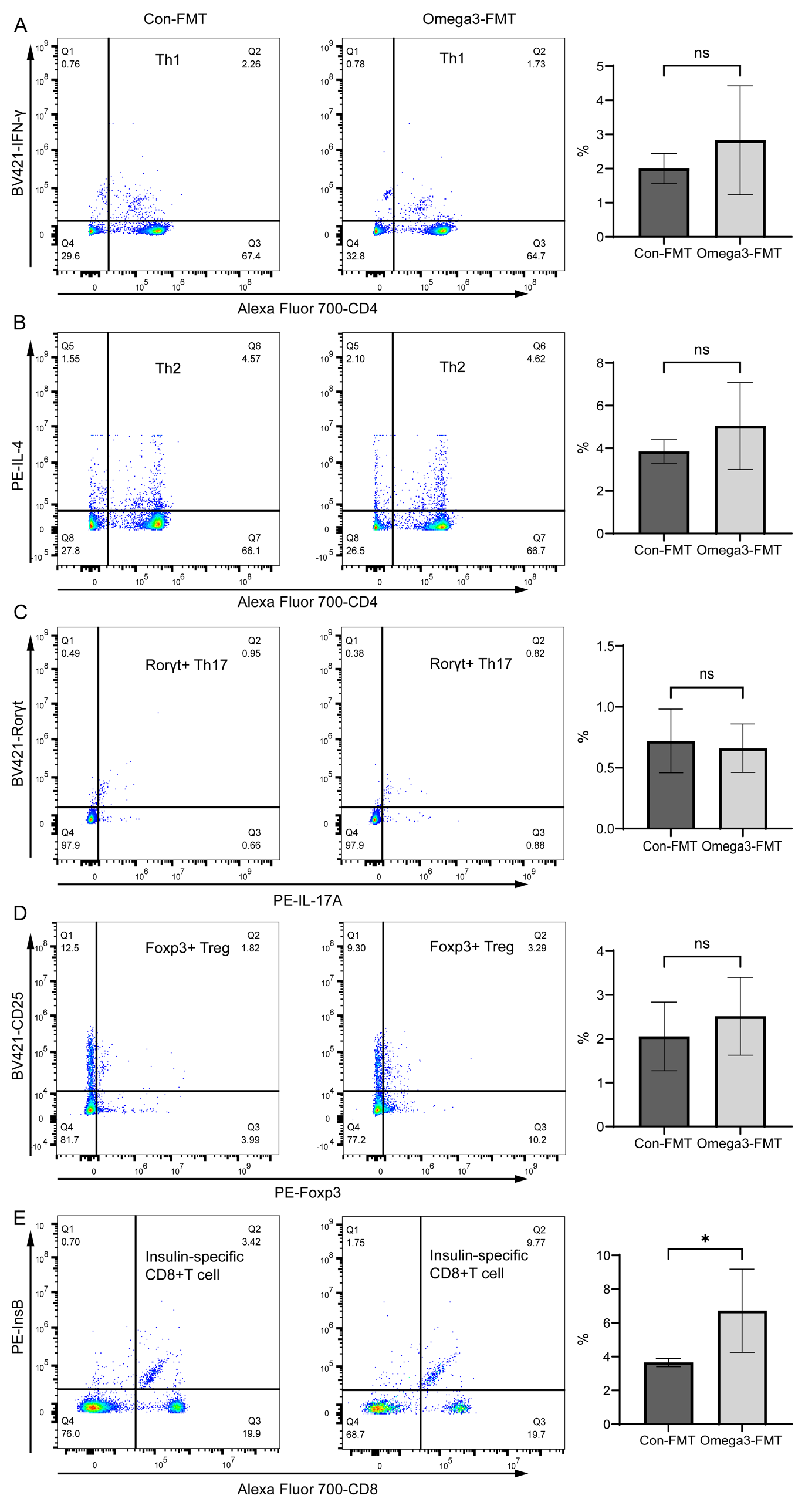

Supplement: Supplementary file 2 [file Image3.tif]

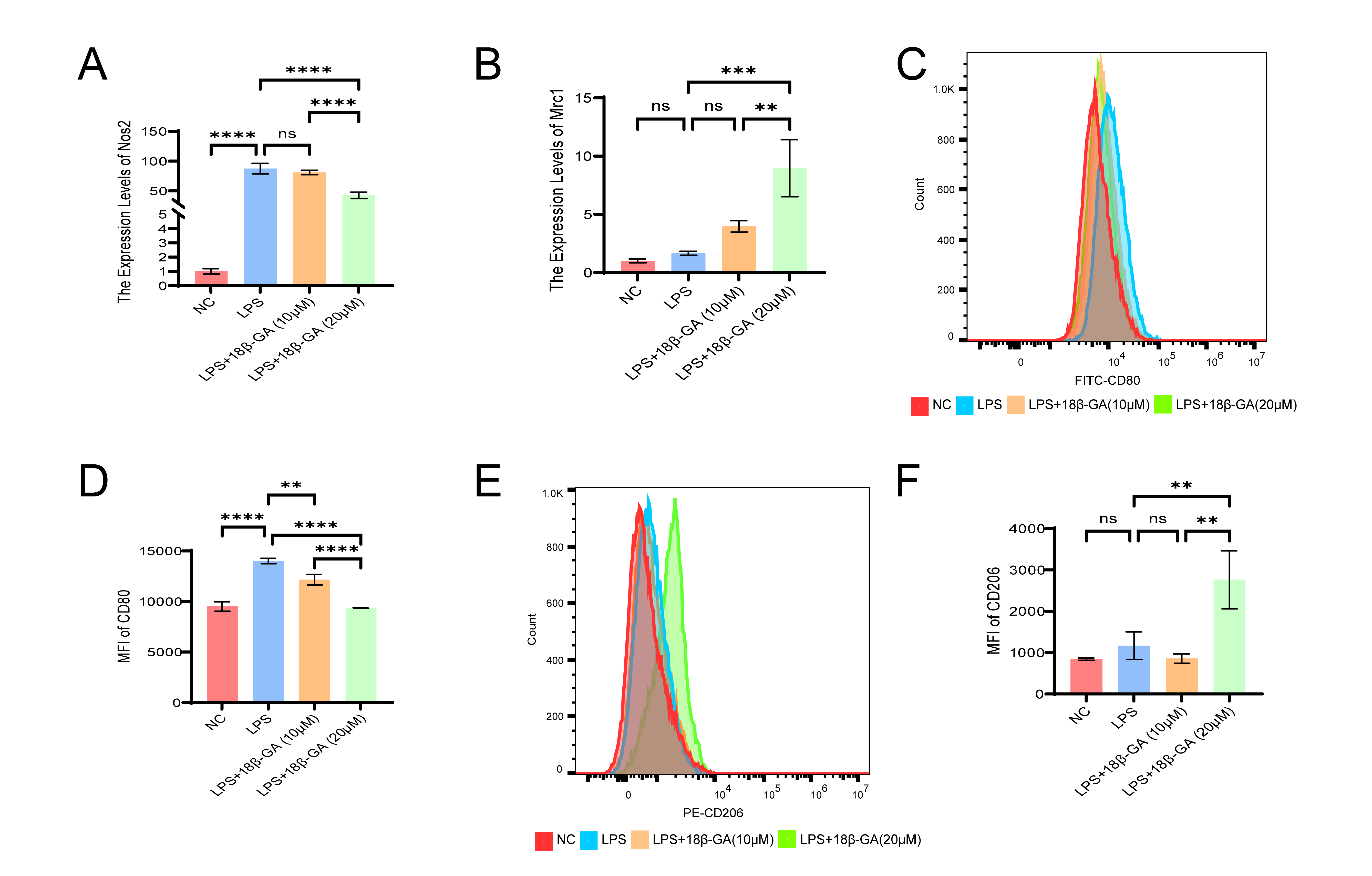

Supplement: Supplementary file 3 [file Image4.tif]

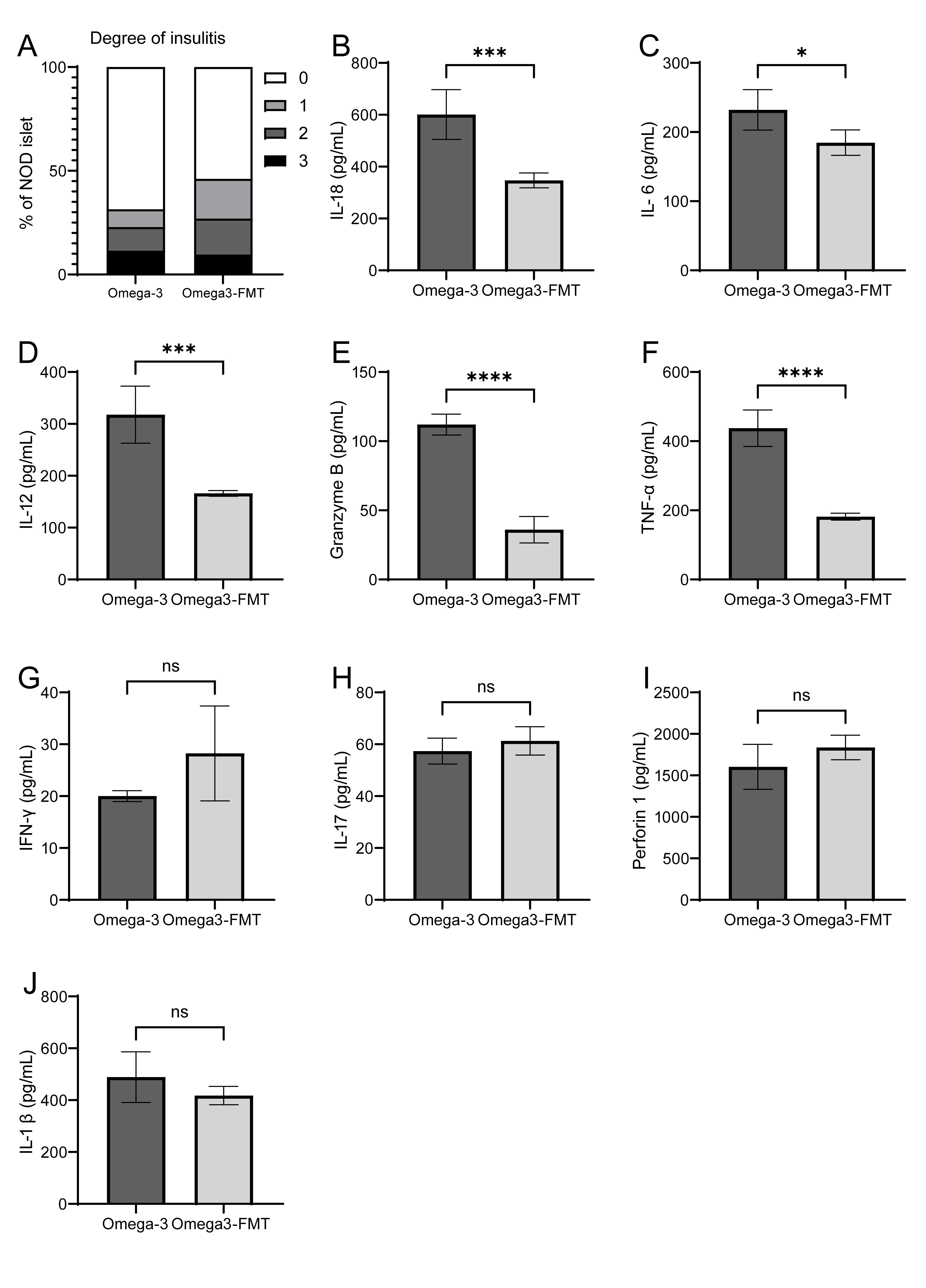

Supplement: Supplementary file 4 [file Image2.tif]

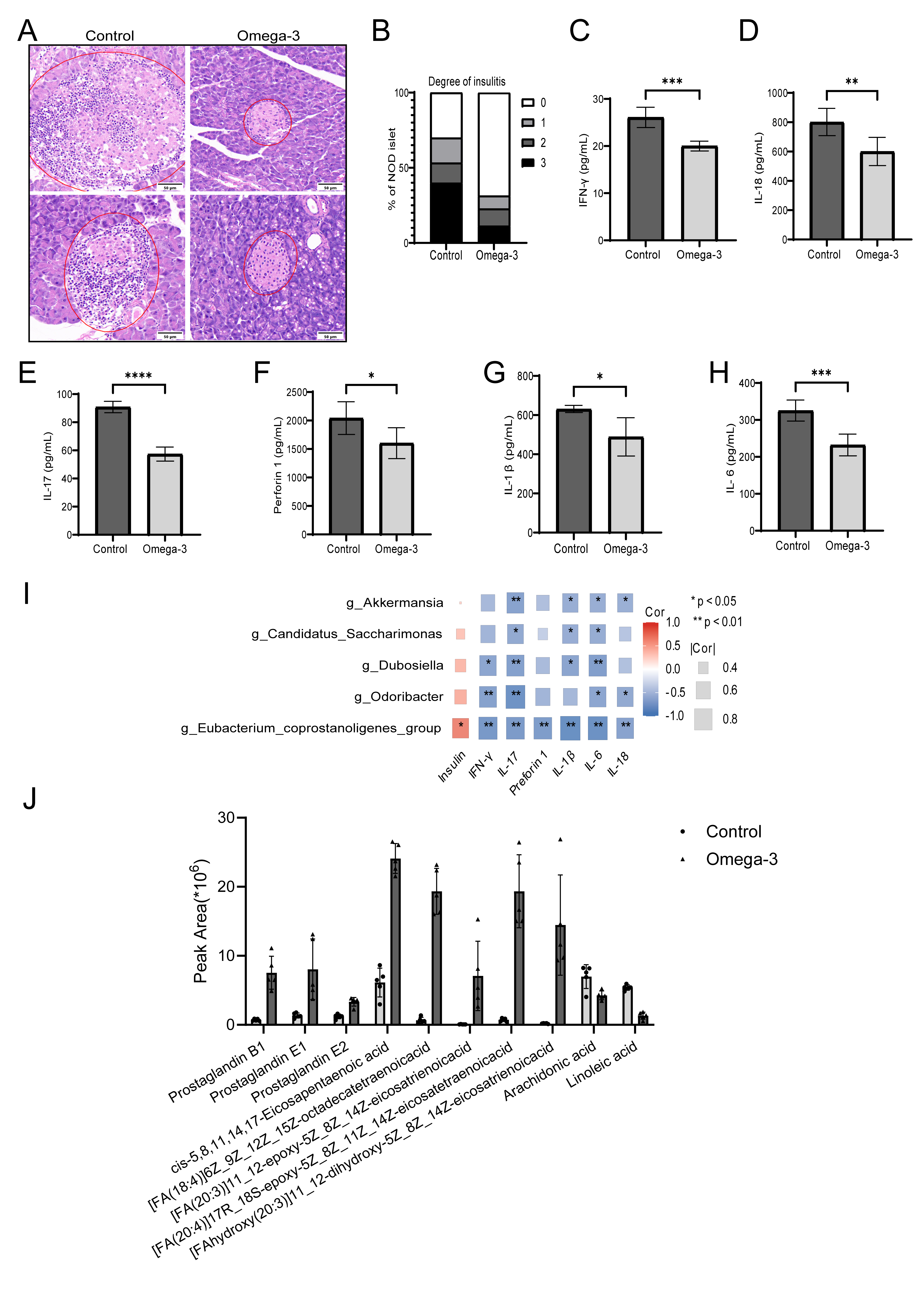

Supplement: Supplementary file 5 [file Image1.tif]
